# Drug–Drug Interaction Study to Evaluate the Pharmacokinetics, Safety, and Tolerability of Ipatasertib in Combination with Darolutamide in Patients with Advanced Prostate Cancer

**DOI:** 10.3390/pharmaceutics14102101

**Published:** 2022-10-01

**Authors:** Dhruvitkumar S. Sutaria, Grozdana Rasuo, Adam Harris, Ryan Johnson, Dale Miles, Jorge Daniel Gallo, Rucha Sane

**Affiliations:** 1 Genentech, Inc., 1 DNA Way, South San Francisco, CA 94080, USA; 2 F. Hoffmann-La Roche AG, 4070 Basel, Switzerland

**Keywords:** pharmacokinetics, drug–drug interaction, CYP metabolism, Akt inhibitor, androgen receptor inhibitor, ipatasertib, darolutamide

## Abstract

Ipatasertib is a selective, small molecule Akt inhibitor that is currently being developed for the treatment of metastatic castration-resistant prostate cancer. Darolutamide is an androgen receptor (AR) inhibitor that is approved for the treatment of non-metastatic castration-resistant prostate cancer. Ipatasertib is metabolized by CYP3A4 to form a less active metabolite M1 (G-037720). Ipatasertib is also a weak time-dependent CYP3A4 inhibitor. Darolutamide is a mild CYP3A4 inducer and is metabolized into an active keto-darolutamide metabolite via CYP3A4. In this Phase 1b open-label, single sequence crossover study, ipatasertib pharmacokinetics safety and tolerability were evaluated in combination with darolutamide in metastatic castration-resistant prostate cancer (*n* = 15 patients). Specifically, the effect of 600 mg BID of darolutamide on 400 mg QD ipatasertib was evaluated in this study. Based on pharmacokinetic analysis, a mild reduction in ipatasertib AUC_0–24 h,ss_ and C_max,ss_ exposures was observed (~8% and ~21%, respectively) when administered in combination with darolutamide, which is considered not clinically meaningful. M1 exposures were similar with and without darolutamide administration. Darolutamide and keto-darolutamide exposures in combination with ipatasertib were similar to previously reported exposures for single agent darolutamide. Overall, the combination appears to be well-tolerated in the metastatic castration-resistant prostate cancer indication with very few AEs.

## 1. Introduction

Prostate cancer is characterized by its dependency on the AR signaling and frequent activation of the Phosphoinositide 3-kinase (PI3K)/protein kinase B (AKT) signaling. Oncogenic activation of the PI3K/AKT pathway either by activating mutations in PI3K, AKT genes or loss of function alterations in tumor suppressor phosphatase and tensin homolog (PTEN) are frequent events in prostate cancer [1]. A preclinical study has demonstrated that in the presence of PTEN deficiency, the AR and PI3K/AKT pathways cross-regulate each other by reciprocal feedback i.e., the inhibition of the first activates the second and vice versa, thereby maintaining cancer cell survival [2]. This reciprocal cross-talk between the PI3K/AKT and AR pathways suggests that the combined inhibition of the AR and PI3K/AKT pathways may be necessary to achieve more complete tumor control and improve efficacy. Preclinical studies conducted using a PTEN-deficient murine prostate cancer model and human prostate cancer xenografts showed that inhibiting both the PI3K/AKT and AR pathways had profound tumor regression compared to inhibiting a single pathway [2]. The combined inhibition of these two pathways by ipatasertib and enzalutamide showed a significant antitumor response in various prostate-specific xenograft models [3].

Ipatasertib is a potent, highly selective small molecule inhibitor of all three isoforms of the AKT. Ipatasertib is shown to be rapidly absorbed with a median time to maximum concentration (T_max_) of 0.5–3 h across 25–800 mg doses in patients with cancer [4]. Ipatasertib exhibited low human plasma protein binding, and the median half-life (t_1/2_) (range) of ipatasertib was 45.0 h (27.8–66.9 h) for oral doses in the range of 200–800 mg. Ipatasertib’s steady-state after administration of a 400 mg QD dose is reached within 7 days of dosing [4]. Ipatasertib is a substrate of CYP3A4 and P-glycoprotein (P-gp) and has shown to be metabolized into a 3–5-fold less active M1 (G-037720) metabolite [4]. Ipatasertib, when combined with itraconazole, a strong CYP3A4 and P-gp inhibitor, increased ipatasertib exposure (AUC_0–__∞_ and C_max_) by 5.45-fold and 2.26-fold, respectively [5]. Ipatasertib is currently being evaluated in metastatic castration-resistant prostate cancer (mCRPC) in combination with abiraterone (a second-generation androgen receptor selective inhibitor) and prednisone/prednisolone. A dedicated drug–drug interaction study conducted to evaluate the effect of abiraterone on ipatasertib showed ipatasertib and M1 exposures were increased by approximately 1.5- and 2.2-fold, respectively, when combined with abiraterone; however, the DDI mechanism is not likely due to CYP3A4 inhibition [6]. Increased incidences of adverse events (AEs) were observed in the ipatasertib–abiraterone–prednisone group compared to the placebo group due to increased ipatasertib exposures and overlapping toxicities [7].

The combination of enzalutamide, a strong CYP3A4 inducer, with ipatasertib resulted in an approximately 50% decrease in ipatasertib exposure, which may result in reduced ipatasertib efficacy [8,9]. Darolutamide is a novel second-generation non-steroidal AR antagonist with greater affinity and no agonist activity for the AR [10]. Darolutamide is a mild CYP3A4 inducer and a CYP3A4 substrate metabolized into an active metabolite, keto-darolutamide. Darolutamide C_max_ is reached approximately 4 h after the administration of a single 600 mg oral dose. The effective t_1/2_ of darolutamide and keto-darolutamide is approximately 20 h in patients. A steady state for darolutamide after administration of 600 mg BID is achieved within 2–5 days of dosing [11,12,13]. Darolutamide has several advantages over other androgen receptor selective inhibitors (ARSIs), such as abiraterone, enzalutamide, and apalutamide, which makes it a good combination partner for ipatasertib. It has a more favorable toxicity profile than other ARSIs as evident from a lower rate of AEs [10]. Darolutamide has a lower penetration of the blood–brain barrier than enzalutamide and apalutamide. Darolutamide has also been reported to have a low potential for clinically relevant DDIs with drugs that are substrates for CYP450 or P-glycoprotein [14,15].

The goal of this clinical trial was, thus, to evaluate the drug–drug interaction and the safety and tolerability of ipatasertib in combination with darolutamide in metastatic and non-metastatic castration-resistant prostate cancer to enable its drug development in prostate cancer.

## 2. Materials and Methods

### 2.1. Study Design and Treatment

**Figure 1:**
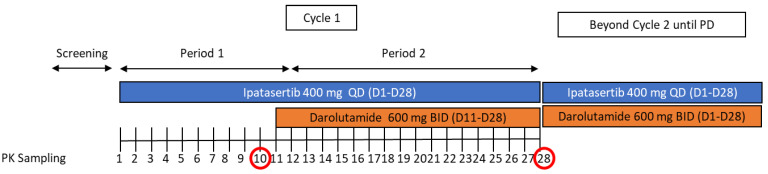
Schematic representation of the study design.

This was an open-label, multicenter, Phase Ib study designed to assess the safety, tolerability, and the drug–drug interaction of the combined administration of 400 mg QD oral ipatasertib and 600 mg BID oral darolutamide. The study was performed in Moldova and Ukraine where 15 patients with metastatic CRPC were enrolled in the study; however, the intended population to be enrolled in this study were both non-metastatic and metastatic CRPC patients. For this study, no prospective calculations of the statistical power were made; the sample size selected was based on a typical sample size used for DDI studies [16]. In this study, ipatasertib 400 mg QD monotherapy was dosed from Cycle 1, Day1 to Cycle 1, Day 10 with or without food, as ipatasertib exposures following a high-fat meal were comparable to that under fasted conditions [17]. From Cycle 1, Day 11 onwards, ipatasertib 400 mg QD was dosed in combination with darolutamide 600 mg BID with food since darolutamide bioavailability is increased by 2.0- to 2.5-fold when administered with food [11,12,13]. Figure 1 presents an overview of the study design.

The primary objectives of the study were to characterize the effect of darolutamide on the PK of ipatasertib and its M1 (G-037720) metabolite when administered alone versus in combination with darolutamide and to evaluate the safety and tolerability of the ipatasertib–darolutamide combination. The PK endpoints of the study were to evaluate the plasma PK parameters of ipatasertib and its M1 metabolite and evaluate the PK of darolutamide and its metabolite keto-darolutamide at a steady state of the combination treatment and compare this to historical data. Medications known to impact the PK of ipatasertib or darolutamide (both CY3PA4 substrates) were refrained from being administered during the study conducted and accordingly not co-administered. The safety endpoints of the study were to evaluate the incidences, nature, and severity of AEs and laboratory abnormalities, changes in vital signs, exposure to the study treatment, and to evaluate the changes in ECG parameters with particular focus on the QT interval corrected through the use of Fridericia’s formula (QTcF). The study achieved the first patient first visit on 21 December 2020, and the cut-off date for primary PK and safety analysis presented within this manuscript was 31 August 2021.

### 2.2. Safety Plan

The safety and tolerability assessments consisted of monitoring and recording AEs, including SAEs and AEs of special interest, clinical laboratory assessments, vital signs, a 12 lead ECG, and physical examination. The AEs were recorded from the first admission until the discontinuation of visits. The AE severity was graded from Grade 1 to Grade 5 as per the National Cancer Institute Common Terminology Criteria for Adverse Events version 5.0. The relationship between the AEs and the study drug was indicated as related or not related. The AE terms reported were coded using the standard Medical Dictionary for Regulatory Activities version 24.0’s preferred terms. In order to manage any drug-related toxicities, ipatasertib and darolutamide modifications were specified as follows: An ipatasertib dose could be reduced in decrements of 100 mg up to 200 mg (can be reduced to two dose levels) beyond which the patient must discontinue ipatasertib but may continue treatment with darolutamide. In the case of darolutamide, the dose could be reduced from 600 mg BID to 300 mg BID until symptoms improve. Treatment may then be resumed at a dose of 600 mg, twice daily. A dose reduction below 300 mg BID for darolutamide was not recommended.

### 2.3. Pharmacokinetic Analysis

Plasma concentrations of ipatasertib and its metabolite M1 were determined using a previously reported, validated liquid chromatography–tandem mass spectrometry assay (LC-MS/MS) [5]. The lower and upper limit of the quantitation for ipatasertib and M1 was 0.500 ng/mL and 400 ng/mL, respectively. Stable labeled internal standards were used for both analytes. Plasma concentrations of darolutamide and keto-darolutamide were determined by a validated bioanalytical assay using supported liquid extraction and LC-MS/MS detection by Labcorp Early Drug Development Laboratories Inc. (Madison, WI, USA). The LC-MS/MS analysis was carried out using an Xbridge C18 column (3.5 mm, 50 × 2.1 mm) on a Shimadzu LC instrument interfaced with a Sciex API 4000 mass spectrometer operated in positive electrospray ionization mode. Gradient elution chromatography utilizing 0.2% formic acid in water (mobile phase A) and 0.2% formic acid in acetonitrile (mobile phase B) was employed to achieve baseline separation of the darolutamide and keto-darolutamide. At a flow rate of 0.450 mL/min, the gradient elution program was initiated at 35% MP B and was gradually increased to 60% over 2.40 min. From 2.40 to 2.60 min, the MP B was ramped to 95% and was maintained until 3.20 min. From 3.20 to 3.8 min, the MP B was returned to the starting conditions, where the system was equilibrated until 4.40 min. The total cycle time from one injection to the next was 5.0 min. The diastereomers of darolutamide, (S, R)-darolutamide, and (S, S)-darolutamide, were quantified as one entity from a single chromatographic peak. The MS/MS transitions monitored for detection were m/z 399.2 to 178.1 for darolutamide, m/z 397.1 to 136.0 for keto-darolutamide, and 478.2 to 221.0 for the internal standard apalutamide. The lower and upper limit of quantitation for darolutamide was 5 ng/mL and 5000 ng/mL, respectively. The lower and upper limit of quantitation for keto-darolutamide was 10 ng/mL and 10,000 ng/mL, respectively. Dilution integrity was validated for samples containing up to 25,000 ng/mL darolutamide and 50,000 ng/mL keto-darolutamide.

PK non-compartmental analysis was performed using the commercial software Phoenix WinNonlin (Certara USA, Inc., Princeton, NJ, USA, Version 8.1). PK samples for ipatasertib and M1 at a steady state were collected at pre-dose, 1, 2, 3, 4, 6, 8, 12, and 24 h post-dose on Cycle 1, Day 10 and Cycle 1, Day 28 to evaluate ipatasertib and M1 exposures in the absence and presence of darolutamide, respectively. PK samples for darolutamide and keto-darolutamide were collected at a steady state at pre-dose, 1, 2, 3, 4, 6, 8, and 12 h post-dose on Cycle 1, Day 28 only.

### 2.4. Statistical Analysis

Descriptive statistics (number of patients, mean, SD, %CV, median, min, max, geometric mean, and geometric %CV) were summarized for all the calculated PK parameters for ipatasertib, M1, darolutamide, and keto-darolutamide. Only median, min, and max were evaluated for Tmax.

Pharmacokinetic parameters (area under the curve (AUC_0–tau_) and C_max,ss_) were compared between Day 10 of Cycle 1 (ipatasertib alone, as the reference treatment) and Day 28 of Cycle 1 (combination of ipatasertib and darolutamide, as the test treatment) using SAS Version 9.4 by calculating geometric mean ratios and corresponding 90% confidence intervals (CIs) to evaluate the effect of darolutamide on the PK of ipatasertib and M1. A linear mixed model with a fixed effect for treatment and a random effect for patients was used on the natural log-transformed pharmacokinetic parameters. Point estimates for the means and point estimates and corresponding 90% confidence intervals (CIs) for the differences in means between the two treatments (reference and test treatments) were obtained from the linear mixed effects model and then exponentiated to obtain geometric means, geometric mean ratios, and respective 90% CIs on the original scale.

## 3. Results

### 3.1. Subjects

Fifteen male patients were enrolled in this study with a median age of 69.0 years (range: 56 years to 79 years) and with a median BMI of 31.50 kg/m^2^ (range: 23.8 kg/m^2^ to 40.6 kg/m^2^). All patients were male, white, and of non-Hispanic or non-Latino ethnicity. All patients had an ECOG Grade of 0 or 1 (Table 1).

### 3.2. Ipatasertib Pharmacokinetics

**Figure 2:**
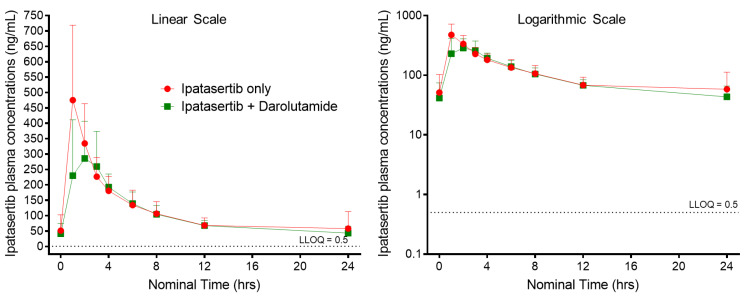
Arithmetic Mean ± SD concentration–time profiles of ipatasertib presented as both linear and logarithmic scales following multiple-dose oral administration of ipatasertib 400 mg QD alone and in combination with darolutamide 600 mg BID.

The mean ipatasertib plasma concentration–time profiles for 400 mg QD ipatasertib alone and in combination with 600 mg BID darolutamide are presented in Figure 2.

Table 2 and Table 3 provide a summary of ipatasertib PK parameters and the statistical analysis of the effect of darolutamide on the PK parameters for ipatasertib, respectively.

Overall, ipatasertib AUC_0–tau_ exposure was comparable between ipatasertib monotherapy and ipatasertib when administered in combination with darolutamide (Table 2). The geometric mean AUC_0–tau,ss_ (geo%CV) exposures for ipatasertib when administered as monotherapy was 2665 h·ng/mL (31.2%) compared to 2399 h·ng/mL (19.6%) when ipatasertib was administered in combination with darolutamide. A mild reduction in C_max,ss_ exposures was observed when ipatasertib was administered in combination with darolutamide compared to ipatasertib monotherapy. The geometric mean (geo%CV) C_max,ss_ exposures for ipatasertib when administered as monotherapy was 462 ng/mL (51.0%) compared to 355 ng/mL (39.8%) when administered in combination with darolutamide (Table 2). Statistical analysis to evaluate the effect of darolutamide on ipatasertib PK showed that ipatasertib exposures were marginally reduced when ipatasertib was coadministered with darolutamide compared to when ipatasertib was administered alone (Table 3). The geometric LS means ratios (90% confidence intervals [CIs] for ipatasertib AUC_0–tau,ss_ and C_max,ss_ were 0.92 (0.84, 1.01) and 0.79 (0.62, 0.99), respectively. The peak plasma concentrations (T_max_) for ipatasertib when administered as monotherapy were achieved with a median time of 1.04 h (range: 1.00 h to 6.10 h). The T_max_ when ipatasertib was administered in combination with darolutamide was slightly delayed with a median time of 2 h (range: 1.00 h to 3.00 h) (Figure 2).

### 3.3. M1 (G-037720) Pharmacokinetics

**Figure 3:**
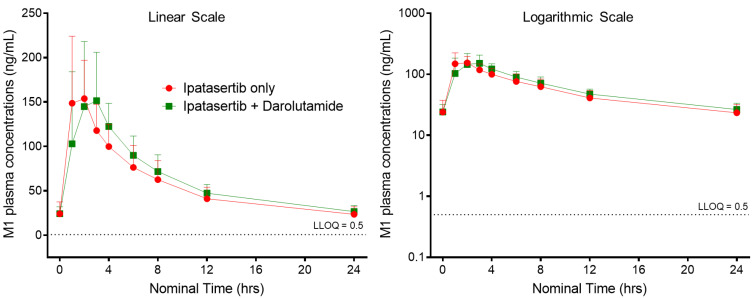
Arithmetic Mean ± SD concentration–time profiles of M1 (G-037720) presented as both linear and logarithmic scales following multiple-dose oral administration of ipatasertib 400 mg QD alone and in combination with darolutamide 600 mg BID.

M1, a pharmacologically less active major metabolite of ipatasertib, was also evaluated in this study. The mean M1 metabolite plasma concentration–time profiles for 400 mg QD ipatasertib alone and in combination with 600 mg BID darolutamide are presented in Figure 3. Table 2 and Table 3 provides a summary of M1 PK parameters and the statistical analysis of the effect of darolutamide on PK parameters for M1, respectively.

M1 AUC_0–tau,ss_ and C_max,ss_ exposures were comparable when ipatasertib was administered alone versus in combination with darolutamide (Table 2). The geometric mean (geo%CV) AUC_0–tau_ exposures for M1 when administered as monotherapy was 1329 h·ng/mL (27.6%) compared to 1475 h·ng/mL (21.7%) when ipatasertib was administered in combination with darolutamide. The geometric mean (geo%CV) C_max,ss_ exposures for M1 (G 037720) when ipatasertib was administered as monotherapy was 169 ng/mL (36.1%) compared to 177 ng/mL (41.1%) when administered in combination with darolutamide (Table 2). Statistical analysis showed that both AUC_0–tau,ss_ and C_max,ss_ of the M1 exposures were comparable when ipatasertib was administered alone and in combination with darolutamide. The geometric LS means ratios (90% CIs) for M1 AUC_0–tau_ and C_max,ss_ were 1.09 (1.03, 1.16) and 1.04 (0.87, 1.25), respectively. T_max,ss_ for M1 metabolite was delayed when ipatasertib was administered in combination with darolutamide (median 2.00 h [range: 1.00 h to 4.00 h]) compared to when ipatasertib was administered as monotherapy (median 1.10 h [range: 1.00 h to 4.10 h]) (Figure 3).

### 3.4. Darolutamide and Keto-Darolutamide Pharmacokinetics

A summary of the PK parameters for darolutamide and keto-darolutamide following a multiple-dose administration of ipatasertib in combination with darolutamide is presented in Table 4.

Darolutamide AUC_0–tau_ and C_max,ss_ exposures when administered in combination with ipatasertib are similar to the reported exposures for single-agent darolutamide [11,12,13]. The darolutamide geometric mean (geo%CV) AUC_0–tau,ss_ exposures in combination with ipatasertib is 51.0 μg·h/mL (26.8%) compared to single-agent darolutamide AUC_0–12 h,ss_ exposure of 52.82 μg·h/mL (33.9%). The darolutamide geometric mean (geo%CV) C_max,ss_ exposures in combination with ipatasertib is 5.43 µg/mL (27.0%) compared to single-agent darolutamide C_max,ss_ exposure of 4.79 (30.9%) μg/mL. Keto-darolutamide AUC_0–tau,ss_ and C_max,ss_ exposures when administered in combination with ipatasertib are similar to the reported exposures for single-agent darolutamide [11,12,13]. The keto-darolutamide geometric mean (geo%CV) AUC_0–tau,ss_ exposures in combination with ipatasertib is 77.0 μg·h/mL (32.7%) compared to that following single-agent darolutamide at AUC_0–12 h,ss_ exposure of 82.8 μg·h/mL (39.5%). The keto-darolutamide geometric mean (geo%CV) C_max,ss_ exposures in combination with ipatasertib is 8.41 μg/mL (28.6%) compared to that following single-agent darolutamide at C_max,ss_ exposure of 9.4 μg/mL (35.4%). Overall, it was observed that both darolutamide and keto-darolutamide exposures in combination with ipatasertib were similar to the reported exposures for darolutamide when administered as a single agent.

### 3.5. Safety Results

In this study, a dosing regimen of single agent ipatasertib 400 mg once daily (QD) from Day 1 to Day 10 and a combination of ipatasertib 400 mg QD and darolutamide 600 mg twice daily (BID) thereafter was administered to patients with mCRPC. When receiving ipatasertib as monotherapy from Day 1 to Day 10 in Cycle 1, two patients (14.3%) reported five AEs of which only one patient reported AEs that were deemed related to ipatasertib. When receiving ipatasertib in combination with darolutamide from Cycle 1 Day 11 onwards, eight patients (57.1%) reported 39 AEs of which four were deemed related to ipatasertib, and no AEs were related to darolutamide. The summary of AEs is presented in Table 5.

When ipatasertib was administered as monotherapy, only one patient (7.1%) reported an AE of diarrhea Grade 1; however, when ipatasertib was administered in combination with darolutamide, one patient (7.1%) had asthenia of Grade 1; two patients (14.3%) had a COVID-19-infection of Grade 1; three patients (21.4%) reported diarrhea of Grade 1; one patient (7.1%) experienced anemia of Grade 2; one patient (7.1%) showed increased levels of alanine aminotransferase of Grade 1; one patient (7.1%) had hyperglycemia of Grade 2; one patient (7.1%) had elevated blood cholesterol levels of Grade 2; two patients had nausea: one (7.1%) of Grade 1 and the other of Grade 2, and one patient (7.1%) experienced vomiting of Grade 1. During the duration of this safety analysis, only two patients experienced SAEs of which one SAE of COVID-19 pneumonia was reported to have led to the interruption of both ipatasertib and darolutamide, and one SAE of platelet reduction was reported to have led to ipatasertib discontinuation. No deaths were reported during the duration of this ongoing study.

There were also no safety signals related to the vital signs observed in the study population. The summary of 12-lead ECG values (heart rate, QT interval, and QTcF) and changes from the baseline did not show any clinically relevant trends; none of the patients had QTcF values that were greater than 60 msec, and only one patient showed a shift in QTcF value of >30 and ≤60 ms.

## 4. Discussion

Ipatasertib is primarily metabolized by CYP3A4 into a 3–5-fold less active M1 metabolite. A concomitant administration of itraconazole, a strong CYP3A4 and P-gp inhibitor, with 100 mg ipatasertib increased ipatasertib exposures (AUC_0–__∞_ and C_max_) by 5.45-fold and 2.26-fold, respectively [5]. In vitro studies have shown ipatasertib to be a time-dependent inhibitor of CYP3A4. Ipatasertib at 600 mg QD increased midazolam, a sensitive CYP3A4 substrate AUC_0–__∞_ exposures, by 2.22-fold [17]. Based on physiological-based pharmacokinetic modeling simulations performed with a clinical ipatasertib dose of 400 mg dose QD, ipatasertib is expected to be a mild CYP3A4 inhibitor and can increase midazolam exposure (AUC_0–__∞_) by approximately 1.69-fold [18]. Ipatasertib, when combined with enzalutamide, a strong CYP3A4 inducer, showed that ipatasertib exposures were decreased by approximately 50% [8].

Darolutamide is a novel AR antagonist approved for the treatment of non-metastatic castration-resistant prostate cancer. Darolutamide is a mild CYP3A4 inducer. A concomitant administration of darolutamide and midazolam reduced midazolam exposures by ~29% [14]. Darolutamide is also a CYP3A4 substrate that is metabolized into an active metabolite (keto-darolutamide). Darolutamide AUC_0–__∞_ and C_max_ exposures were increased by 1.7-fold and 1.4-fold, respectively, when combined with itraconazole.

It has been reported that midazolam’s exposures were reduced by approximately 85% when it is administered in combination with enzalutamide, a strong CYP3A4 inducer [19]. Based on the observed effect of enzalutamide and darolutamide on midazolam exposures, and extrapolating it to ipatasertib, it was expected that ipatasertib exposures would likely be reduced only marginally when administered in combination with darolutamide. As both compounds are CYP3A4 substrates and at the same time affect CYP3A4 activity, this study evaluated the PK DDI potential between ipatasertib and darolutamide, as well as the safety of the combination. Overall, this study confirmed a mild drug interaction between ipatasertib and darolutamide. The effect of a 600 mg darolutamide treatment administered twice daily in combination with 400 mg ipatasertib administered once daily was, as expected, a mild interaction showing only a slight reduction of ipatasertib AUC_0–tau,ss_ and C_max,ss_ exposures. The geometric LS mean ratios (90% CIs) for ipatasertib AUC_0–tau,ss_ and C_max,ss_ were 0.92 (0.84, 1.01) and 0.79 (0.62, 0.99), respectively. The exposures of the ipatasertib M1 metabolite was similar when ipatasertib 400 mg QD was co-administered with darolutamide 600 mg BID. The geometric LS mean ratios (90% CIs) for M1 AUC_0–tau,ss_ and C_max,ss_ were 1.09 (1.03, 1.16) and 1.04 (0.87, 1.25), respectively.

Darolutamide and keto-darolutamide exposures in combination with ipatasertib were similar to previously reported exposures for single-agent darolutamide, suggesting no interaction of ipatasertib on the pharmacokinetics of darolutamide. Based on the observed pharmacokinetic DDI results from this study, only a mild reduction was observed for ipatasertib exposure, and no changes were observed for darolutamide and keto-darolutamide, which suggests a low potential for a drug–drug interaction overall. These clinical results are in line with what was expected based on the observed effect of darolutamide on midazolam exposures, and extrapolating it to ipatasertib, which is a less sensitive CYP3A4 substrate compared to midazolam. In addition, the safety results showed that the combination was well tolerated, and there were no safety signals related to the vital signs observed in the study population; however, these results need to be interpreted with caution given the small number of patients in this study.

## 5. Conclusions

Overall, the combination of ipatasertib 400 mg QD administered with darolutamide 600 mg BID appears to be generally well-tolerated in non-metastatic and metastatic CRPC with very few AEs reported. In summary, darolutamide appears to be a good combination partner with ipatasertib for further evaluation in prostate cancer.

## Figures and Tables

**Table 1:** Summary of demographics and baseline characteristics of patients enrolled in this study.

Parameter	Category/Statistic	No of Subjects ^a^ (%)
Sex, *n* (%)	Male	15 (100%)
Indication	Metastatic Castration-Resistant Prostate Cancer	15 (100%)
	Non-Metastatic Castration-Resistant Prostate Cancer	0
Race	White	15 (100%)
	Black of African American	0
	Asian	0
	American Indian or Alaska Native	0
	Native Hawaiian or other Pacific Islander	0
	Other	0
Ethnicity	Hispanic or Latino	0
	Not Hispanic or Latino	15 (100%)
Age (y)	Median (range)	69.0 (56–79)
Weight (kg)	Mean (range)	92.55 (73.0–123.0)
Height (cm)	Mean (range)	173.0 (161.0–189.0)
BMI (kg/m^2^)	Mean (range)	31.01 (23.8–40.6)
ECOG	0	10 (66.7%)
	1	5 (33.3%)

^a^ Age, weight, height, and BMI are indicated as ranges.

**Table 2:** Summary of ipatasertib and M1 (G-037720) pharmacokinetics following multiple-dose administration of ipatasertib 400 mg QD alone and in combination with darolutamide 600 mg BID.

	PK Parameter	Ipatasertib (*n* = 14)	Ipatasertib with Darolutamide (*n* = 13) *
Ipatasertib	AUC_0–tau,ss_ (h·ng/mL)	2665 (31.2)	2399 (19.6)
	AUC_0–t,ss_ (h·ng/mL)	2666 (31.1)	2399 (19.6)
	C_max,ss_ (ng/mL)	462 (51.0)	355 (39.8)
	T_max,ss_ (h)	1.04 (1.00, 6.10)	2.00 (1.00, 3.00)
M1 (G-037720)	AUC_0–tau,ss_ (h·ng/mL)	1329 (27.6)	1475 (21.7)
	AUC_0–t_ (h·ng/mL)	1329 (27.6)	1475 (21.7)
	C_max,ss_ (ng/mL)	169 (36.1)	177 (41.1)
	T_max,ss_ (h)	1.10 (1.00, 4.10)	2.00 (1.00, 4.00)

AUC_0–tau,ss_ = area under the plasma concentration–time curve from t = 0 up to t = tau post-dose at a steady state; AUC_0–t,ss_ = area under the plasma concentration–time curve from time 0 up to t hours post-dose (i.e., the last measurable concentration) at a steady state; C_max,ss_ = maximum observed plasma concentration in a steady state; *n* = number of evaluable subjects; PK = pharmacokinetic; tau = 24 h for ipatasertib; T_max,ss_ = time to attain C_max,ss_; Geometric means (%CV) are presented for AUC_0–tau,_ AUC_0–t,ss,_ and C_max,ss_; median (min, max) are presented for T_max,ss_; * One subject did not receive an ipatasertib dose on Day 28 of Cycle 1 and was, thus, excluded from descriptive statistics for Cycle 1 Day 28 timepoint.

**Table 3:** Statistical analysis results of ipatasertib and M1 (G-037720) metabolite pharmacokinetic parameters following multiple-dose administration of ipatasertib 400 mg QD alone and in combination with darolutamide 600 mg BID.

		Test		Reference		Geometric LS Mean Ratio (Test/Ref)	
Analyte	PK Parameter	*n*	Geometric Mean	*n*	Geometric Mean	Estimate	90% CI
Ipatasertib	AUC_0–tau,ss_ (ng·h/mL)	13	2399	13	2609	0.92	(0.84, 1.01)
	C_max,ss_ (ng/mL)	13	355	13	452	0.79	(0.62, 0.99)
M1 (G-037720)	AUC_0–tau,ss_ (ng·h/mL)	13	1475	13	1349	1.09	(1.03, 1.16)
	C_max,ss_ (ng/mL)	13	177	13	170	1.04	(0.87, 1.25)

AUC_0–tau,ss_ = area under the plasma concentration–time curve from time 0 up to t = tau post-dose at a steady state; C_max,ss_ = maximum observed plasma concentration at a steady state; CI = confidence interval; PK = pharmacokinetic; LS = least squares; tau = 24 h for ipatasertib. ‘Reference’ and ‘Test’ are ‘Ipatasertib only’ and ‘Ipatasertib with Darolutamide’.

**Table 4:** Summary of darolutamide and keto-darolutamide pharmacokinetics following multiple-dose administration of ipatasertib 400 mg QD in combination with darolutamide 600 mg BID.

	PK Parameter	Ipatasertib with Darolutamide	Single-Agent Darolutamide from the Literature (11–13)
Darolutamide (*n* = 14)	AUC_0–tau,ss_ (h·µg/mL)	51.0 (26.8)	-
	AUC_0–t,ss_ (h·µg/mL)	51.7 (27.0)	52.82 (33.9)
	C_max,ss_ (µg/mL)	5.43 (27.0)	4.79 (30.9)
	T_max,ss_ (h)	4.23 (2.00, 8.25)	-
Keto-darolutamide (*n* = 14)	AUC_0–tau,ss_ (h·µg/mL)	77.0 (32.7)	-
	AUC_0–t,ss_ (h·µg/mL)	78.0 (33.5)	82.8 (39.5)
	C_max,ss_ (µg/mL)	8.41 (28.6)	9.4 (35.4)
	T_max,ss_ (h)	4.13 (1.00, 8.25)	-

AUC_0–tau,ss_ = area under the plasma concentration–time curve from time 0 up to t = tau post-dose at a steady state; AUC_0–t,ss_ = area under the plasma concentration–time curve from time 0 up to t hours post-dose (i.e., the last measurable concentration) at a steady state; BID = twice daily; C_max,ss_ = maximum observed plasma concentration in a steady state; *n* = number of evaluable subjects; PK = pharmacokinetic; QD = once daily; tau = 12 h for darolutamide; T_max,ss_ = time to attain C_max_. Geometric means (%CV) are presented for AUC_0–tau,ss_, AUC_0–t,ss,_, and C_max,ss_; median (min, max) are presented for T_max._

**Table 5:** Summary of adverse events when ipatasertib 400 mg QD is administered alone and ipatasertib 400 mg QD is administered in combination with darolutamide 600 mg BID.

AE Category	Ipatasertib (N = 14) *n* (%)	Ipatasertib with Darolutamide (N = 14) *n* (%)
Total number of patients with at least one AE	2 (14.3)	8 (57.1)
Total number of AE events	5	39
Total number of deaths	0	0
Total number of patients with at least one		
Any treatment discontinuation	0	1 (7.1)
AE leading to discontinuation of ipatasertib	0	1 (7.1)
AE leading to dose reduction of ipatasertib	0	1 (7.1)
AE leading to dose interruption of ipatasertib	0	2 (14.3)
AE leading to discontinuation of darolutamide	0	1 (7.1)
AE leading to dose reduction of darolutamide	0	0
AE leading to dose interruption of darolutamide	0	2 (14.3)
Grade ≥ 3 AE	0	2 (14.3)
Grade 5 AE	0	0
Serious AE	0	2 (14.3)
AE related to any treatment	1 (7.1)	4 (28.6)
AE related to ipatasertib only	1 (7.1)	4 (28.6)
AE related to darolutamide only	0	0

AE = adverse event; N = number of subjects who reported AEs; *n* = percent of subjects; Multiple occurrences of the same AE in one individual are counted only once except for ‘Total number of AE events’ row in which multiple occurrences of the same AE are counted separately.

## Data Availability

Qualified researchers may request access to individual patient-level data through the clinical study data request platform (https://vivli.org/). Further details on Roche’s criteria for eligible studies are available here (https://vivli.org/members/ourmembers/). For further details on Roche’s Global Policy on the Sharing of Clinical Information and how to request access to related clinical study documents, see here (https://www.roche.com/research_and_development/who_we_are_how_we_work/clinical_trials/our_commitment_to_data_sharing.html).
